# Cycle Inhibiting Factors (Cifs): Cyclomodulins That Usurp the Ubiquitin-Dependent Degradation Pathway of Host Cells

**DOI:** 10.3390/toxins3040356

**Published:** 2011-03-29

**Authors:** Frédéric Taieb, Jean-Philippe Nougayrède, Eric Oswald

**Affiliations:** 1 INRA, USC Molecular and Cellular Pathogenesis of *Escherichia coli* Infections, Toulouse, F-31300, France; Email: jp.nougayrede@envt.fr (J.-P.N.); e.oswald@envt.fr (E.O.); 2 Inserm, U1043, Toulouse, F-31300, France; 3 University of Toulouse, UPS, Centre de Physiopathologie de Toulouse Purpan (CPTP), Toulouse, F-31300, France; 4 CNRS, U5282, Toulouse, F-31300, France; 5 CHU Toulouse, Hôpital Purpan, Service de Bactériologie-Hygiène, Toulouse, F-31300, France

**Keywords:** bacterial toxin, type III secretion system, eukaryotic cell cycle, NEDD8, ubiquitin, cullin-RING E3 ubiquitin ligase

## Abstract

Cycle inhibiting factors (Cifs) are type III secreted effectors produced by diverse pathogenic bacteria. Cifs are “cyclomodulins” that inhibit the eukaryotic host cell cycle and also hijack other key cellular processes such as those controlling the actin network and apoptosis. This review summarizes current knowledge on Cif since its first characterization in enteropathogenic *Escherichia coli*, the identification of several xenologues in distant pathogenic bacteria, to its structure elucidation and the recent deciphering of its mode of action. Cif impairs the host ubiquitin proteasome system through deamidation of ubiquitin or the ubiquitin-like protein NEDD8 that regulates Cullin-Ring-ubiquitin Ligase (CRL) complexes. The hijacking of the ubiquitin-dependent degradation pathway of host cells results in the modulation of various cellular functions such as epithelium renewal, apoptosis and immune response. Cif is therefore a powerful weapon in the continuous arm race that characterizes host-bacteria interactions.

## 1. Discovery and Distribution of the Cycle Inhibiting Factors (Cifs) That Triggers an Original Cytopathic Effect in Host Cells

**Figure 1:**
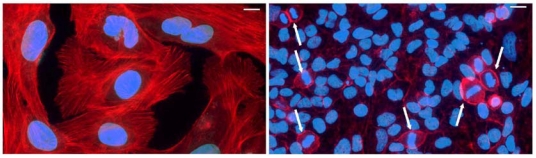
Cycle inhibiting factor (Cif) induces cell enlargement, actin stress fibers and proliferation arrest. HeLa cells were infected with enteropathogenic *Escherichia coli* (EPEC) producing Cif (left panel) or Cif mutant EPEC (right panel). F-actin is stained with phalloidin (red) and DNA is stained with DAPI (blue). Arrows indicate mitotic/dividing cells. Bars represent 20 µM.

**Figure 2:**
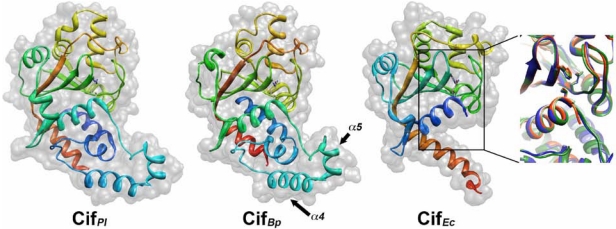
Cif homolog proteins are structurally related and share a conserved catalytic site. Cif*_Pl_*, Cif*_Bp_* and Cif*_Ec_* are represented as composites of ribbon and surface plot diagrams. Note that the crystallized Cif*_Ec_* protein lacked 99 amino acids in its *N*-terminal part. The side chains of the critical residues of the catalytic triad (Cys, His, Gln) are shown. The α-helices α4 and α5 of Cif*_Bp_* are indicated. The right panel represents a superimposition of the three Cifs magnified at the level of the catalytic pocket.

The effect of Cif was first observed in 1997 by De Rycke *et al.* in HeLa cells transiently infected with enteropathogenic *Escherichia coli* (EPEC) strains isolated from weaning rabbits or human infants with diarrhea [1]. This so-called cytopathic effect (CPE) was first characterized by the progressive formation of actin stress fibers together with focal adhesions, and a dramatic cell enlargement (Figure 1). It was further shown that these large cells were irreversibly impaired for cell proliferation, with a complete lack of mitotic figures [2] (Figure 1). This effect is dependent on the locus of enterocyte effacement (LEE), the cluster of genes responsible for the attaching and effacing lesion, the hallmark of EPEC and enterohemorraghic *E. coli* (EHEC) infection [3,4]. The protein responsible for the CPE identified by Marches *et al.* was called Cif (cycle inhibiting factor). The gene coding for Cif is located outside the LEE, on a lambdoid prophage [5]. 

Two epidemiological studies showed that 60 to 70% of EPEC and EHEC strains are *cif*-positive [6,7]. However, in one of these studies, it was shown that two thirds of these *cif*-positive *E. coli* strains do not induce a typical CPE due to frameshift mutations or insertion of an IS element in the *cif* gene. Strikingly, *cif* is always found as a pseudogene in EHEC strains; this could be due to the small size of the strain collection, or could suggest an evolutionary counter-selection of functional Cif in Shiga-like producing EHEC strains [6]. Nonetheless, plasmid-mediated expression of wild type Cif in EHEC confers the CPE phenotype [5].

The *E. coli* Cif protein is 282 amino-acids long, showing no similarity with any protein except several homologues in the human pathogens *Yersinia pseudotuberculosis* (56% of identity) and beta-proteobacterium *Burkholderia pseudomallei* (26%). Cif homologues are also found in the entomopathogens *Photorhabdus luminescens* (23%) and *Photorhabdus asymbiotica* (26%). The Cif proteins are thus named Cif*_Ec_*, Cif*_Yp_*, Cif*_Bp_*, Cif*_Pl_* and Cif*_Pa_* respectively. In all these bacteria the *cif* genes are located in prophage or prophage-like region (*Escherichia* and *Photorhabdus* species), bordered by two vestigial transposase genes (*Burkholderia*) and in a locus frequently rearranged nearby the *yrs* region homologous to a recombination target of bacteriophages (*Yersinia*) [8]. Thus Cif-encoding regions are found in DNA domains prone to rearrangement, suggesting that the *cif* genes were acquired by horizontal gene transfer. Consistent with this hypothesis, the Cif-producing EPEC strain E22 was shown to produce *cif*-carrying infectious phage particles supporting that the *cif* gene was disseminated by horizontal gene transfer among EPEC and enterohemorrhagic (EHEC) strains, as shown for other non LEE-encoded effectors [6,9,10].

Analysis of the Cif xenologues showed that they are all functional as they reproduced the typical Cif*_Ec_*-associated CPE when transfected into HeLa cells, or lipofected following purification [8]. Importantly, Cif*_Pl_* was shown to be expressed *in vivo* in an insect model, the natural host of *Photorhabdus*, and reproduced the CPE in the non-mammalian insect Sf9 cell line *in vitro* [11]. Similarly, Cif*_Bp_* is also active *in vitro *when strains of *E. coli* (EPEC) or *Burkholderia thailandensis *are transformed with a plasmid coding for Cif*_Bp _*and used to infect mammalian cell lines [8,12]. Such conservation of an effector function among very distant pathogens belonging to β-protobacteria and γ-protobacteria that infect invertebrate and mammal hosts is unique and remarkable. It also supports the role of Cifs as virulence factors and could reveal an original strategy of pathogenesis. However, *Photorhabdus* spp. are symbiotic of the nematode vector *Heterorhabditis* (that releases the bacteria into the insect) suggesting that Cif might also act as a fitness or symbiotic factor in the nematode host.

## 2. Cif Proteins are Type III Effectors That Traffic to the Nucleus of the Host Cells

Initial experiments showed that the CPE triggered by *cif*-positive EPEC strains was dependent on the type III secretion system (T3SS) encoded by the LEE as mutants of the secretion and translocation apparatus (EscN and EspA, EspB, EspD, respectively) were unable to induce the CPE [1,2,5,13]. This T3SS injects an arsenal of effector proteins into the host cell that hijack cellular functions for the pathogen’s benefit [3,4,14]. Using a reporter system based on a translational fusion of the effector protein with β-lactamase that allows detection of translocation in living host cells, Charpentier and Oswald showed that an exchangeable *N*-terminal translocation signal comprising the first 16 amino acids of Cif*_E__c_* is necessary and sufficient to mediate its translocation by the EPEC T3SS [15]. Using a lipid-based cell delivery system bypassing T3SS requirement, purified Cif*_Ec_* induced a CPE similar to that observed upon EPEC infection. This result indicated that Cif*_Ec_* is necessary and sufficient and does not require other bacterial molecules to induce the CPE [16]. Once injected into the host cell cytoplasm by the T3SS, Cif*_Ec_* is rapidly sorted to the peri-nuclear area and the nucleus, as observed by confocal imaging and cell fractionation [17]. As Cif*_Ec_* lacks classical nuclear localization sequences, the mechanism of nuclear targeting of Cif remains to be elucidated.

While Cif*_Bp_* has been shown to be injected by the T3SS of *Escherichia* and *Burkholderia* species and successfully reproduces the CPE [8,12], we and other have been unable to translocate Cif*_Yp_* and Cif*_Pl_* by the *E. coli* T3SS into the host cells. However, we have indirect evidence that they are *bona fide* type 3 effectors as both *Yersinia* and *Photorabdhus* strains possess T3SS. Moreover, none of the Cifs could induce the CPE without electroporation, transfection or lipofection suggesting that Cif activity depends on its injection into the host cell [8,11,16]. 

## 3. Crystal Structure Studies of Cif Homologs Reveals a Family of Proteins Sharing a Conserved Active Site

Despite a low level of sequence similarity between Cif homologs, the crystal structure determination of Cif*_Ec_*, Cif*_Pl _*and Cif*_Bp_* showed that these proteins are structurally well-conserved (Figure 2) [18,19,20]. The overall structure of these Cif can be divided in two lobes with head and tail domains where the *N*-terminal part corresponds to the tail and contains the potential substrate binding site (see below) whereas the *C*-terminal part forms the head domain hosting the enzymatic site. The enzymatic site consists in a catalytic triad composed of residues Cys, His and Gln strictly conserved in all Cifs homologs (Figure 2).

The conserved catalytic triad is located in a negative charged cleft with a putative occluding loop near the active site that might control the substrate accessibility to the catalytic site [19]. The structural organization of this triad reveals that Cifs are divergent members of the superfamily of enzymes that comprises papain-like cysteine proteases, acetyl transferases, deamidases and transglutaminases. In particular, Cif shares homology with the avirulence effector AvrPphB from *Pseudomonas syringae* that belongs to the same superfamily and with the cysteine protease YopT from *Yersinia* spp. [19]. Mutation of one of the residues of the catalytic triad abolishes Cif*_Pl_*, Cif*_Bp_*, Cif*_Ec_* and Cif*_Yp_* activity, corroborating the implication of an enzymatic-dependent activity [8,16,19,20]. Further analysis of the tail domain showed that deletion of the *N*-terminal protruding α4 and α5 helix of Cif*_Bp_* (Figure 2) and of the corresponding α4 domain of Cif*_Ec_* suppressed the toxin activity suggesting that this domain could mediate substrate recognition [17,20].

Based on the structure and function conservation, Cif homologs constitute a family of cyclomodulins that likely target a molecule/pathway conserved in a wide range of eukaryotic cells from invertebrates to mammals [8,21].

## 4. Cifs Are Cyclomodulins That Trigger Host Cell Cycle Arrest

Upon injection into host cells by the T3SS (or by transfection or lipofection of purified proteins) Cif proteins induce an irreversible cell cycle arrest with complete inhibition of mitotis entry (Figure 1: absence of mitotic figures in cells injected with Cif). This property makes Cifs as members of the cyclomodulin family defined as bacterial molecules that hijack host cell cycle functions [22,23].

**Figure 3:**
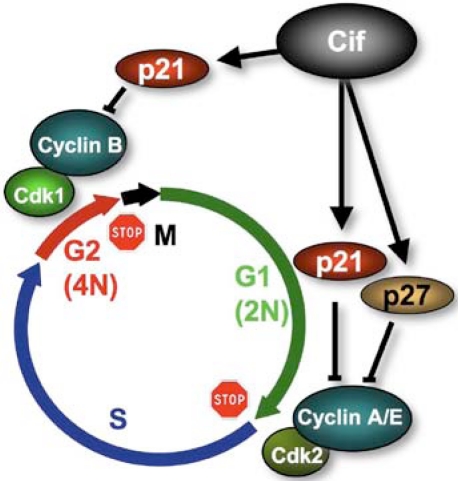
Cif inhibits host cell cycle progression. Cif induces the accumulation of p21 and p27 that inhibits CDK1-CyclinB and CDK2-CyclinA/E. Since these complexes are responsible for G2/M and G1/S transitions, Cif provokes an accumulation of cells arrested in G1 and G2 phases of the cell cycle.

The cell cycle arrest induced by Cif*_Ec_* is associated to the inhibitory phosphorylation of the cyclin dependent kinase (CDK) 1-CyclinB complex whose activation is necessary for the cell cycle G_2_/M transition [1,2,5]. Other cyclomodulins, such as CDT or Colibactin, also block the G_2_/M transition through inhibition of CDK1 activity. However, in contrast to CDT and Colibactin, which have been shown to be *bona fide* genotoxins, Cif*_Ec_* does not induce DNA damage nor activate the DNA damage response pathway (ATM/ATR, CHK1/2, CDC25 sequestration) that leads to the inhibitory phosphorylation of CDK1 [16]. Therefore, Cif*_Ec_* induces the cell cycle arrest by activating a pathway independent from the canonical activation of the G_2_/M checkpoint. Further studies, using synchronized cells, have shown that Cif*_Ec_* also blocks S-phase entry. Depending on the stage of the cell cycle during infection with *cif*-positive EPEC, cells arrest either at the G_1_/S or G_2_/M transition. The analysis of host cell proteins regulating both S- and M- phases entries demonstrated a Cif-dependent accumulation of p21^Waf1/Cip1^ and p27^kip2^ (hereafter called p21 and p27) [24]. These proteins belong to the Cip/Kip family of CDK inhibitors (CKI). p21 is an inhibitor of the CDK1/CyclinB complex. This complex is the eukaryotic universal inducer of mitosis entry and p21 accumulation is known to inhibit G_2_/M transition. Both p21 and p27 inhibit CDK2-CyclinE and A complexes whose activation are also required for G1/S and S-phase progression. p21 also binds to PCNA, and the latter is required for S-phase progression [25]. Therefore, Cif-dependent accumulation of CKI is consistent with the observed G_1_/S and G_2_/M cell cycle arrests (Figure 3). The inhibitory phosphorylation of CDK1 observed upon Cif*_Ec_* injection could be explained by the disruption of the CDK1 positive feed-back loop leading to CDK1 dephosphorylation via CDC25. Further investigation revealed that CKI accumulation does not depend on transcriptional activation but relies on their stabilization in infected cells [24]. Since p21 and p27 are degraded by the ubiquitin-proteasome system, these results strongly suggested that Cif*_Ec_* controls this proteolytic pathway. However, suppression of p21 and/or p27 accumulation did not alleviate Cif-induced inhibition of G_1_/S or G_2_/M transitions implying an alternative mechanism for blockage of the cell cycle [24]. Whether this arrest is associated to stress fibers induction was excluded since disruption of stress fiber formation in HeLa cells by C3 exoenzyme-mediated inhibition of RhoA, did not alleviate cell cycle inhibition [2]. Morevover, only HeLa and RK13 cells exhibit stress fibers following *cif* ^+^ EPEC infection suggesting that these two phenotypes are not related [16]. Nonetheless, these results indicated that accumulation of CKI participates in a multi-step process leading to the Cif-induced arrest of cell proliferation. 

This cell cycle inhibition and CKI accumulation was observed with all Cif homologues [8] and in various cell lines derived from colonic epithelium (differentiated or not differentiated Caco-2 cells, DLD1, HCT116 wild-type, p21^−/−^ or p53^−/−^) showing the independency of cell-cycle arrest from the p53 transcriptional program [16,24]. The Cif-induced cystostatic effect is also observed in non-transformed IEC-6 cells derived from rat intestinal crypt epithelium. However, the outcome of this arrest is cell type dependent as it persists for more than 3 days in HeLa cells without evident cell death, whereas it culminates in apoptosis 48 h after infection of IEC-6 cells [26]. Whether this Cif-induced cell death is a consequence of the cytostatic effect or relies on an independent pathway is still unclear, but it is tempting to propose that the functionality of p53 pathway, intact in non-transformed cells, might be decisive for the fate of Cif-arrested cells. 

## 5. Cif Effector Proteins Target NEDD8 to Inhibit Specific Ubiquitin-Dependent Degradation Pathways

Three independent teams recently deciphered the molecular mode of action of Cif*_Ec_* and Cif*_Bp_* [12,17,27]. Despite few differences mainly related to the species origin of Cif and diverse approaches, these complementary studies showed a common mechanism of action between the two xenologs.

### 5.1. Cif_Ec_ Interacts with Host Protein NEDD8

**Figure 4:**
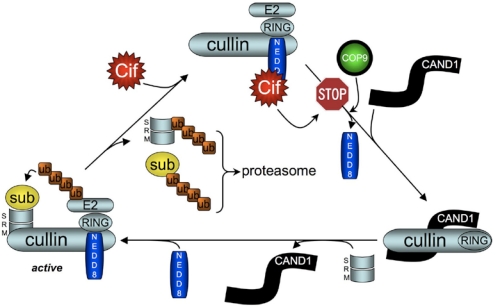
Model of Cif*_Ec_* inhibition of CRL activation cycle. Neddylated active CRL (left) ubiquitylates the substrate (sub) and substrate recognition module (SRM) leading to their degradation and generating inactive CRL (top). Deneddylation of CRL by the COP9 signalosome allows binding of CAND1 (right). SRM fixation induces CAND1 release and subsequent neddylation of cullin to generate an active CRL [35,36]. Cif binding to and deamidation of NEDD8 impairs the action of COP9, blocking the activation cycle and locking the CRL in an inactive neddylated state.

As mentioned earlier, Cif-dependent accumulation of p21 and p27 results from the inhibition of their degradation by the ubiquitin-proteasome system [28]. Ubiquitylation is a well-known and highly controlled post-translational protein modification that involves three successive steps. First, ubiquitin is processed by the ubiquitin activating enzyme (E1) and then transferred to the ubiquitin conjugating enzyme (E2). The last step requires the ubiquitin ligase (E3) that determines the substrate to be ubiquitylated and promotes a direct transfer of ubiquitin from E2 to the substrate (RING-type E3s) or an indirect transfer via formation of an ubiquitin thioester-bound E3 prior ubiquitin transfer to the substrate (HECT-type E3s). Repetitions of this cycle lead to the attachment of a polyubiquitin chain to the target protein, resulting in modulation of its function or localization, or its sorting for degradation, depending on the length and the type of linkage within the polyubiquitin chain [29,30,31]. Yeast two-hybrid screens and pull-down assays showed that Cif*_Ec_* interacts with the ubiquitin-like protein NEDD8 [17,27]. The main known role of NEDD8 is the conjugation of the cullin subunit of the complex cullin-RING E3 ubiquitin ligase (CRL) and activation of its ligase activity [32,33,34]. CRL are composed of the scaffold protein cullin (at least five different cullins CUL1/2/3/4A/4B are known), the RING protein that associates with E2, and different substrate recognition modules that bind the target protein (Figure 4) [35,36]. Inactivation of Cif*_Ec_* by mutation of the catalytic cysteine increased its binding to NEDD8 [17]. In contrast, deletion of the α4 domain of Cif*_Ec_*, which results in loss of activity, abolished the interaction with NEDD8 implying the role of this domain to mediate Cif effect. Cif*_Ec_* was unable to pull-down ubiquitin showing the specificity of interaction with NEDD8. Moreover, NEDD8 co-compartmentalizes with Cif*_Ec_* in the nuclei of infected cells supporting the functional binding of these 2 proteins [17]. Co-immunoprecipitation experiments showed that Cif*_Ec_* interacts only with the neddylated forms of cullins (CUL1 to 4B) associated with the RING protein [17] and with the recognition module Skp1 and Skp2 proteins [27]. It thus appeared that Cif*_Ec_* interacts with the whole CRL complex through binding to NEDD8 (Figure 4).

### 5.2. Cif Inhibits CRL Activity

Since Cif proteins induce the stabilization of p21 and p27, which are substrates of CRL associated to CUL1, *in vitro* studies were conducted to investigate the inhibitory effect of Cif on CRL. Jubelin *et al.* showed that addition of Cif*_Ec_* to reconstituted CRL inhibited its ubiquitin ligase activity [17]. Moreover, *in cellulo* ubiquitylation of various CRL substrates such as p27 or RhoA was severely impaired by Cif*_Ec_* and Cif*_Bp_*, respectively [12,27]. Stabilization of various CUL1 to 4B-associated CRL substrates including β-catenin, RhoA, IκBα, Cdt1, CyclinD1 reveals that Cif*_Ec_* can interfere with all neddylated cullins [17,27].

How Cif*_Ec_* inhibits CRL activity is not fully elucidated. However, Cui *et al.* showed that the CRL inhibition is associated to Cif*_Ec_*-dependent NEDD8 deamidation of Gln40, converting glutamine residue in glutamic acid (Glu). Replacement of NEDD8 with deaminated NEDD8 (NEDD8Q40E) abolished the *in vitro* stimulatory effect on Nrf2 ubiquitylation by a CUL3/ROC1/Keap1 complex. Similarly, ectopic expression of NEDD8Q40E in HeLa cells resulted in the stabilization of CRL substrate and recapitulated the effects of Cif [12]. On the other hand, Cif*_Ec_* was shown to promote cullin neddylation of CUL1 to 4B [17] that correlates with the inhibition of deneddylation of cullin by the CSN complex (COP9 signalosome) [27]. This phenotype is similar to the effect observed using CSN5 null cells that also accumulate CRL substrates such as p27 [37]. Together, these results suggest that modification of NEDD8 by Cif inhibits cullin deneddylation by the CSN resulting in a decrease of CRL activation. Indeed, to be fully active, CRLs undergo an activation cycle in which cullins oscillate between neddylated and non-neddylated states [35,36]. Based on these results, we propose a model in which Cif*_Ec_* interferes with this cycle and thus locks CRLs in a neddylated but inactive state (Figure 4).

A notable difference between Cif*_Bp_* and Cif*_Ec_* is the capacity of Cif*_Bp_* to deamidate not only NEDD8, but also the Gln40 of ubiquitin. Cif*_Bp_* generates an E2-ubiquitinQ40E thioester complex that impairs ubiquitin chain synthesis and the ubiquitylation pathway *in vitro* and *in vivo* [12]. This supplementary affinity to ubiquitin extends the inhibitory activity of Cif*_Bp_* to all ubiquitylation process independently of the E3 ligase. The relevance of this difference is still unclear and requires further studies on the range of action of other Cif homologs and bacterial deamidases toward ubiquitin and ubiquitin-like molecules. In this line, it is noteworthy that the *Mycobacterium* deamidase of Pup (Dop), required for the full virulence of *M. tuberculosis*, targets the prokaryotic ubiquitin-like molecule Pup to convert the terminal glutamine residue into glutamate [38,39]. Also, while acting through a different pathway to stimulate RhoA activity, it is remarkable that the cyclomodulin CNF-1 directly deamidates RhoA to induces actin stress fibers [40,41].

## 6. Concluding remarks and perspectives

The role of NEDD8 on CRL activation offers to the bacteria injecting Cif into the host cells an “Achilles heel” to hijack various cellular signaling functions. Indeed, CRLs represent the largest subfamily of E3s and therefore play regulatory roles in numerous and diverse cellular functions. The diversity of substrate recognition modules associated to the different cullin subunits gives to the CRL the capacity to ubiquitylate and thus to control the stability of hundreds of proteins [35,36]. Cif capacity to induce stress fibers in certain eukaryotic cells could be related to Cif-stabilization of RhoA [12,17], which is targeted by CUL3-associated CRL [42]. Prevention of inappropriate replication depends on the degradation of the licensing factor Cdt1 during S-phase [43]. As CUL4-associated CRL targets Cdt1 for ubiquitylation-dependent degradation [44,45], inhibition of CRL by Cif provides a satisfactory explanation for occurrence of re-replication observed 3 days following infection as Cdt1 is stabilized in infected HeLa cells [2,16,17]. Moreover, Cif was shown to prevent the degradation of IκB, a central inhibitor of NF-κB pro-inflammatory response [12,17]. Down-regulation of the NF-κB pathway has been proposed to mediate the inflammatory tolerance of commensal bacteria in the mammalian intestinal epithelia [46,47]. In addition, stabilization of β-catenin by Cif might participate in dendritic cells tolerogenicity as it was recently shown that the WNT signaling pathway regulates immunosuppressive responses [48]. 

**Figure 5:**
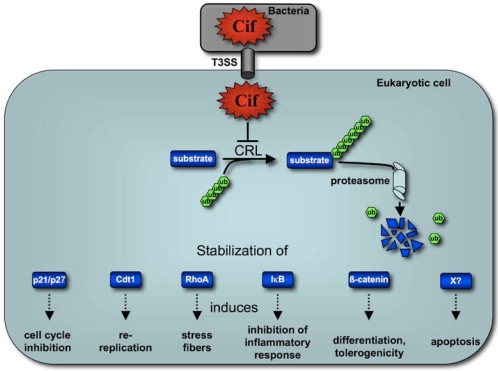
Cif hijacks several and various signaling pathways by inhibiting the ubiquitin ligase activity of CRL. Cif is injected in the host cells and impairs CRL activity via binding to NEDD8. This inhibition results in stabilization of (**i**) p21 and p27 leading to cell cycle inhibition; (**ii**) Cdt1 allowing inappropriate replication; (**iii**) RhoA inducing actin stress fibers; (**iv**) IkB leading to alteration of inflammatory response; (**v**) β-catenin promoting differentiation process disruption and tolerogenicity; (**vi**) unidentified protein(s) inducing apoptosis.

In conclusion, Cif might represent a fitness factor that facilitates bacterial evasion from the host immune response, via inhibition of inflammatory pathways in dendritic cells of the gut-associated lymphoid tissue. Cif-inhibition of the host cell cycle might slow down multiplication of intestinal progenitors to delay epithelial cells renewal, thus favoring gut colonization (Figure 5). We could also speculate about the role of Cif as a stabilizer of other type III co-injected effectors, to modulate their effect on host cells and boost the virulence of *cif*-expressing strains. Further studies of the impact of Cif in the pathogen-host interactions will undoubtedly contribute to our knowledge of bacterial pathogenic strategies. It also appears that the continuous “arm race” that characterizes host-pathogen relationship has lead the bacteria to develop sophisticated “weapons” (virulence factors) capable of controlling host’s functions for their own benefit. Therefore, much can be learned from eukaryotic functions using bacterial toxins such as Cif. While most pharmacological inhibitors or bacterial/viral proteins inhibit CRL by decreasing cullin neddylation [49,50,51,52], Cif is the first example of a bacterial effector that inhibits CRL functions by stabilizing cullin neddylation. Thus Cif represents a unique tool to study the regulation of the ubiquitin proteasome system. Lately, it was reported that inhibitors of NEDD8 represent a promising approach for cancer treatment [52] opening a potential development of innovative therapeutics using Cif-producing bacteria.
